# A MODEL FOR TEACHING STUDENTS AGE-FRIENDLY CONCEPTS USING VIRTUAL INTERPROFESSIONAL SIMULATION

**DOI:** 10.1093/geroni/igad104.2609

**Published:** 2023-12-21

**Authors:** Diane Brown, Cynthia Hovland, Susan Fosnight, Brandi Chrzanowski, Mary Gergis, Rikki Patton, Darcia Simpson

**Affiliations:** University of Akron, Akron, Ohio, United States; Cleveland State University, Cleveland, Ohio, United States; Summa Health System, Akron, Ohio, United States; Direction Home Akron Canton Area Agency on Aging & Disabilities, Uniontown, Ohio, United States; Cleveland State University, Cleveland, Ohio, United States; Drexel University, Philadelphia, Pennsylvania, United States; Hospice of the Western Reserve, Cleveland, Ohio, United States

## Abstract

Achieving effective Age-Friendly Health Systems requires teaching the future healthcare workforce how to consistently use the 4Ms framework in the care of older adults: What Matters, Medication, Mentation, and Mobility. Effective interprofessional teamwork is key to operationalizing this model. We describe here a virtual interprofessional student simulation training that demonstrates the importance of teamwork in addressing each of the 4Ms concepts and, importantly, how inattention to one ‘M’ can lead to ineffective care. Our training prepares learners with online didactic pre-work followed by a virtual simulation experience where interprofessional groups of students simulate a team care planning meeting. The case demonstrates how ‘what matters most’ to the patient (i.e., providing care for her husband who has dementia) impacts each of the other Ms. Specifically, she is taking medications inappropriately, has symptoms of depression, and will not undergo recommended surgery to correct mobility issues due to concerns about who will care for her husband. We have conducted this training with 548 undergraduate and graduate students representing medicine, nursing, pharmacy, social work, nutrition/dietetics, counseling, physical, occupational and speech therapy. 95% of students rated the simulation experience positively. There was statistically significant improvement in appreciation for teamwork (p< 0.001), evidence-based practice (p< 0.001), and inclusion of the patient and caregiver perspectives (p< 0.001) comparing pre and post simulation survey results. These results indicate a positive impact on student’s preparedness to incorporate 4Ms principles into their future practice, thus advancing both Age Friendly and Dementia Friendly Health System initiatives.

